# The asparagine 533 residue in the outer pore loop region of the mouse PKD2L1 channel is essential for its voltage‐dependent inactivation

**DOI:** 10.1002/2211-5463.12273

**Published:** 2017-08-14

**Authors:** Takahiro Shimizu, Taiga Higuchi, Toshihiro Toba, Chie Ohno, Takuto Fujii, Bernd Nilius, Hideki Sakai

**Affiliations:** ^1^ Department of Pharmaceutical Physiology Graduate School of Medicine and Pharmaceutical Sciences University of Toyama Japan; ^2^ Laboratory of Ion Channel Research Department of Cellular and Molecular Medicine KU Leuven Belgium

**Keywords:** channel, inactivation, PKD2L1, transient receptor potential, TRPP3

## Abstract

Voltage‐dependent inactivation of ion channels contributes to the regulation of the membrane potential of excitable cells. Mouse polycystic kidney disease 2‐like 1 (PKD2L1) forms voltage‐dependent nonselective cation channels, which are activated but subsequently inactivated in response to membrane depolarization. Here, we found that the mutation of an asparagine 533 residue (N533Q) in the outer pore loop region of PKD2L1 caused a marked increase in outward currents induced by depolarization. In addition, the tail current analysis demonstrated that the N533Q mutants are activated during depolarization but the subsequent inactivation does not occur. Interestingly, the N533Q mutants lacked the channel activation triggered by the removal of stimuli such as extracellular alkalization and heating. Our findings suggest that the N533 residue in the outer pore loop region of PKD2L1 has a key role in the voltage‐dependent channel inactivation.

**Abbreviations**
CSF‐c: cerebrospinal fluid contacting
HERG: human ether‐à‐go‐go‐related gene
KcsA: *Streptomyces lividans* K^+^ channel
PKD2L1: PKD2‐like 1
PKD2: polycystic kidney disease 2
TRPP3: TRP polycystin 3
TRP: transient receptor potential
*V*_half_: half‐maximal activation voltage
WT: wild‐type

Transient receptor potential (TRP) proteins constitute a superfamily of nonselective cation channels. The TRP protein subunit has six transmembrane domains with intracellular N and C termini and a putative pore region between transmembrane segments 5 and 6 1, 2. Polycystic kidney disease 2‐like 1 (PKD2L1) (also called TRP polycystin 3 [TRPP3]) is a member of the TRP superfamily 3. We have so far demonstrated that mouse PKD2L1 functions as a voltage‐dependent nonselective cation channel regulated by extracellular pH and ambient temperature 4, 5, 6, 7. Interestingly, the PKD2L1 channel exhibits unique gating kinetics. Although the cells expressing the PKD2L1 channels show quite small depolarization‐triggered outward currents, large tail currents are observed during the subsequent repolarization. The current response indicates that the PKD2L1 channels can enter an inactivated state. In the gating model, depolarization first activates but then immediately inactivates the PKD2L1 channels, therefore showing small outward currents upon depolarization. The tail currents triggered by the following repolarization represent quick transition of the PKD2L1 channel from inactivated to open states. Thus, the voltage‐dependent inactivation is essential for robust activation of the PKD2L1 channels upon repolarization. The structural basis for the channel inactivation during depolarization, however, is poorly understood.

It is well known that some voltage‐dependent K^+^ channels exhibit two types of voltage‐dependent inactivation, which are referred to as N‐ and C‐type inactivation 8, 9, 10, 11. In N‐type inactivation, the intracellular N‐terminal region of the channel occludes the inner pore. In contrast, C‐type inactivation is associated with structural changes around the selectivity filter of the channel. Among voltage‐dependent K^+^ channels, human ether‐à‐go‐go‐related gene (HERG) K^+^ channels display a characteristic voltage‐dependent gating 12, 13, 14. In response to depolarization, the HERG K^+^ channels first activate but subsequently inactivate. Then, upon repolarization, the inactivated channels are rapidly reactivated. The voltage‐dependent inactivation in the HERG K^+^ channel is considered to be due to C‐type inactivation, because the mutation of amino acid residues adjacent to the selectivity filter of the channel, N629 and S631, suppresses the inactivation 12, 15, 16. As the voltage‐dependent gating of the PKD2L1 channel has some resemblance to that of the HERG K^+^ channel, in the present study, we focused on amino acid residues behind the selectivity filter of the PKD2L1 channel, N531 and N533, to investigate the mechanism of the PKD2L1 channel inactivation.

## Materials and methods

### Site‐directed mutagenesis

**Figure 1:**
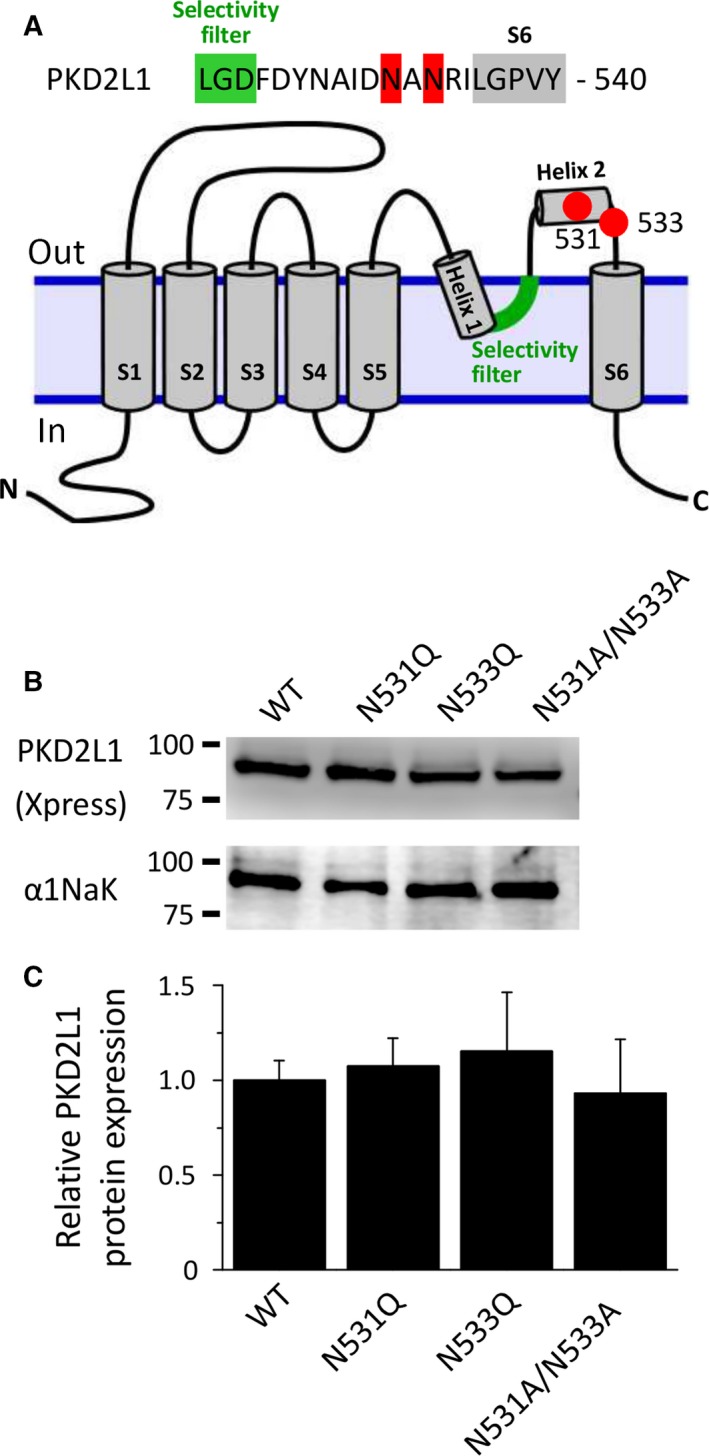
Expression levels of mouse PKD2L1 channels transfected into HEK293T cells. (A) Mutation sites of the PKD2L1 channel. The selectivity filter, the point mutation sites, and transmembrane segment 6 are shown in green, red, and gray, respectively. Membrane topology of the PKD2L1 channel is referred to the three‐dimensional structure of PKD2 channel. (B) Western blotting of membrane fractions from HEK293T cells expressing the wild‐type PKD2L1 (WT) channel and each mutant (N531Q, N533Q, and N531A/N533A). Upper and lower panels show the expression levels of PKD2L1 (~ 90 kDa) and Na^+^,K^+^‐ATPase α1 isoform (α1NaK, 95 kDa), respectively. The α1NaK was used as a loading control. (C) Relative expression levels of the PKD2L1 proteins. The data are averaged from four independent experiments.

Several point mutants in the outer pore loop region of the mouse PKD2L1 channel (N531A/N533A, N531Q, N533Q) were constructed (Fig. 1A). The mutations were introduced into the mouse PKD2L1 expression vector (pCINeo‐PKD2L1‐IRES‐GFP) 4, 5, 6 using a Quikchange Lightning Site‐Directed mutagenesis kit (Agilent Technologies, Santa Clara, CA, USA). To generate the double mutant (N531A/N533A), we followed a single‐primer protocol 17 using the primer (aatgccatcgacgctgccgccagaatcctgggccctgtgtactttgtcac). For the N531Q and N533Q mutants, the following primer pairs were used (N531Q, sense: gactacaatgccatcgaccaggccaacagaatcctgggc and anti‐sense: gcccaggattctgttggcctggtcgatggcattgtagtc; N533Q, sense: tgccatcgacaatgcccaaagaatcctgggccctg and anti‐sense: cagggcccaggattctttgggcattgtcgatggca). The sequences of these mutants were verified using an ABI PRISM 3130 genetic analyzer (Applied Biosystems, Foster City, CA, USA).

To quantify expression levels of the PKD2L1 mutants, we introduced a sequence encoding an Xpress epitope [amplified from the pcDNA4/His expression vector (Invitrogen, Carlsbad, CA, USA)] into each mouse PKD2L1 expression vector. The generated vectors were sequenced to confirm the appropriate location of the Xpress epitope.

### Cell culture and transfection

Human embryonic kidney HEK293T cells were grown in Dulbecco's modified Eagle's medium (Sigma‐Aldrich Japan, Tokyo, Japan) supplemented with 10% fetal bovine serum, 100 unit·mL^−1^ penicillin, and 100 μg·mL^−1^ streptomycin at 37 °C in a humidity‐controlled incubator with 5% CO_2_. Each expression vector was transiently transfected into HEK293T cells using Lipofectamine 2000 reagent (Invitrogen). Transfected HEK293T cells were detached from the plastic substrate and cultured on cover slips (Matsunami Glass, Osaka, Japan) before electrophysiological experiments. Patch‐clamp experiments were carried out with GFP‐positive cells 48 h after transfection.

### Electrophysiological experiments

Whole‐cell patch‐clamp recordings were performed using an Axopatch 200B patch‐clamp amplifier (Molecular Devices, Sunnyvale, CA, USA). clampex 9.2 software (Molecular Devices) was used for command pulse control and data acquisition. Currents were filtered at 1 kHz and digitized at 10 kHz. The data were analyzed with clampfit 9.2 (Molecular Devices) and winascd software (kindly provided by G. Droogmans). Patch electrodes had a resistance of 2–4 MΩ when filled with pipette solution. The access resistance was electrically compensated by 70% to minimize voltage errors. To investigate the voltage dependence of PKD2L1 channels, step pulses were applied from −100 to +200 mV in 20‐mV increments with a postpulse to −100 mV. PKD2L1 channel activity (NPo, where N is the number of channels and Po is the open probability) was obtained by dividing each tail current by the corresponding single‐channel current amplitude recorded at −100 mV. The voltage dependence of the channel activity was fitted to the Boltzmann equation.

To analyze single‐channel properties of the PKD2L1 channel, whole‐cell patch membranes were clamped at −60 mV. All‐point amplitude histograms were created from continuous current traces of 30 s. The single‐channel current amplitude was measured as the peak‐to‐peak distance in Gaussian fits of the histograms. The channel activity (NPo) was calculated by dividing the mean current amplitude of each current trace by the corresponding single‐channel amplitude, as previously described 4, 5, 6.

The pipette (intracellular) solution consisted of 130 mm Cs‐aspartate, 2 mm Na_2_ATP, 10 mm MgCl_2_, 1 mm ethylene glycol‐bis(2‐amino ethylester)‐N,N,N’,N’‐tetraacetic acid (EGTA), and 10 mm 4‐(2‐hydroxyethyl)‐1‐piperazineethanesulfonic acid (HEPES), buffered at pH 7.3 with CsOH. The bathing (extracellular) solution contained 130 mm NaCl, 1 mm MgCl_2_, 10 mm HEPES, and 40 mm mannitol, buffered at pH 7.4 with NaOH. To prepare basic bathing solutions, HEPES was replaced with an equal amount of TAPS at pH 9.0 or CAPS at pH 10.0. In some experiments, the temperature of bathing solutions was changed with a temperature controller (TC‐324B, Warner Instruments, Hamden, CT, USA), as previously described 6.

### Western blotting

Western blotting of the HEK293T cells expressing Xpress‐tagged PKD2L1 channels was performed 48 h after transfection. The membrane proteins were prepared as described previously 18, 19, 20. For blotting, mouse anti‐Xpress (Invitrogen) and mouse anti‐Na^+^,K^+^‐ATPase α1 isoform (Santa Cruz Biotechnology, Dallas, TX, USA) antibodies were used in a 1 : 5000 dilution. A HRP‐conjugated donkey anti‐mouse IgG antibody (Merck Millipore, Darmstadt, Germany) was used as a secondary antibody (1 : 5000 dilution). Chemiluminescent signals were quantified using a WesternSure ECL Substrate (LI‐COR Biosciences, Lincoln, NE, USA), a Chemiluminescent Western Blot Scanner (C‐DiGit, LI‐COR Biosciences), and image studio software (LI‐COR Biosciences).

### Statistics

Data are presented as means ± SEM of *n* observations. Statistical differences in the data were evaluated by unpaired Student's *t*‐test or one‐way ANOVA with Tukey's post hoc tests and were considered significant at *P *<* *0.05.

## Results

### Membrane expression of mouse PKD2L1 mutants

Western blotting was performed using membrane fractions prepared from the HEK293T cells overexpressing Xpress‐tagged PKD2L1 proteins. As shown in Fig. 1B, Xpress‐tagged PKD2L1 proteins were detected as a band at expected size (~90 kDa). The expression level of each PKD2L1 mutant was similar to that of the wild‐type (WT) channel (Fig. 1C).

### The outer pore loop region of the mouse PKD2L1 channel regulates the voltage‐dependent inactivation

**Figure 2:**
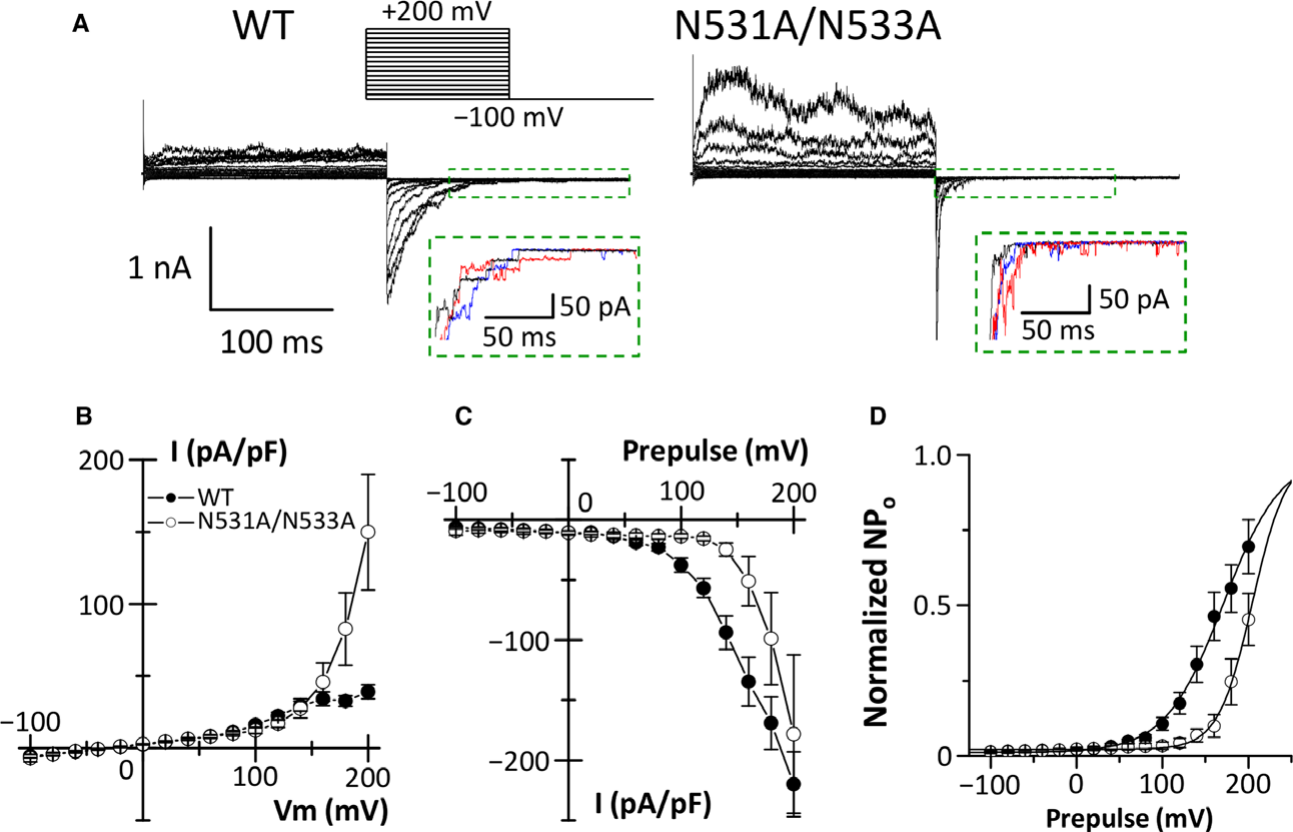
Effects of a double mutation, N531A/N533A, of mouse PKD2L1 on whole‐cell PKD2L1 currents. (A) Representative whole‐cell currents obtained from HEK293T cells expressing the WT channels and the N531A/N533A mutants. The step pulse protocol is shown in the top panel. Insets: Tail currents at −100 mV evoked by prepulses of +160 mV (black), +180 mV (blue), and +200 mV (red) enclosed in a dashed box are enlarged to show single‐channel currents of the PKD2L1 proteins. (B and C) Current–voltage relationships of steady‐state currents (B) and instantaneous tail currents (C) of the WT channels (closed circles) and the double mutants (open circles). (D) Activation curves for the WT channels and the double mutants were obtained as described in Materials and methods. Solid lines represent fits with the Boltzmann equation. The data are averaged from three to eight experiments.

We have previously reported that mouse PKD2L1 channels exhibit voltage‐dependent inactivation 4. Although depolarization‐triggered outward currents of the PKD2L1 channels are small, large tail currents are observed during the subsequent repolarization (see Fig. 2A), suggesting that PKD2L1 channels activate but then immediately inactivate upon depolarization. Here, we investigated whether the outer pore loop region of the PKD2L1 channel is involved in the depolarization‐induced inactivation, based on the functional role of the corresponding region in the HERG K^+^ channel. As we previously had a variety of values for half‐maximal activation voltage (*V*_half_) in the voltage‐dependent activation curve of PKD2L1 channels due to small tail currents 4, 5, 6, in the present experiments, we applied stronger depolarizing pulses up to +200 mV to precisely analyze the voltage‐dependent gating of the PKD2L1 channel (Fig. 2A, inset). HEK293T cells overexpressing the WT channels showed typical PKD2L1 currents; that is, the outward currents triggered by depolarization were small, but larger tail currents were observed upon repolarization to −100 mV (Fig. 2A–C). In comparison with the WT channels, the N531A/N533A double mutants, in which the length of the side chains becomes shorter, exhibited larger outward currents at potentials greater than +160 mV (Fig. 2A,B). The decay of tail currents was accelerated in the cells expressing the N531A/N533A mutants (Fig. 2A). In addition, the current–voltage relationship for the tail currents indicates that the N531A/N533A mutants had a different voltage dependence compared to the WT channels (Fig. 2C). As we could measure the single‐channel PKD2L1 current at the end of the tail currents as shown in Fig. 2A inset, we calculated the PKD2L1 channel activity (NPo), which was obtained by dividing the tail currents by the corresponding single‐channel current amplitude. Figure 2D shows the activation curves for WT channel activity (NPo) and for N531A/N533A mutant activity (NPo). The double mutants showed a rightward shift and steeper slope of the activation curve compared with the WT channels (Fig. 2D). The *V*_half_ was significantly shifted from 163.1 ± 7.3 mV in the WT channels to 204.1 ± 6.7 mV in the N531A/N533A mutants (*P *=* *0.01). The slope factor was statistically different between the WT channels (29.2 ± 1.7 mV) and the N531A/N533A mutants (18.5 ± 0.9 mV, *P *=* *0.02). On the other hand, the single‐channel current amplitude in the N531A/N533A mutants (−19.6 ± 0.6 pA, *P *=* *0.47) was similar to that in the WT channels (−20.6 ± 0.6 pA).

**Figure 3:**
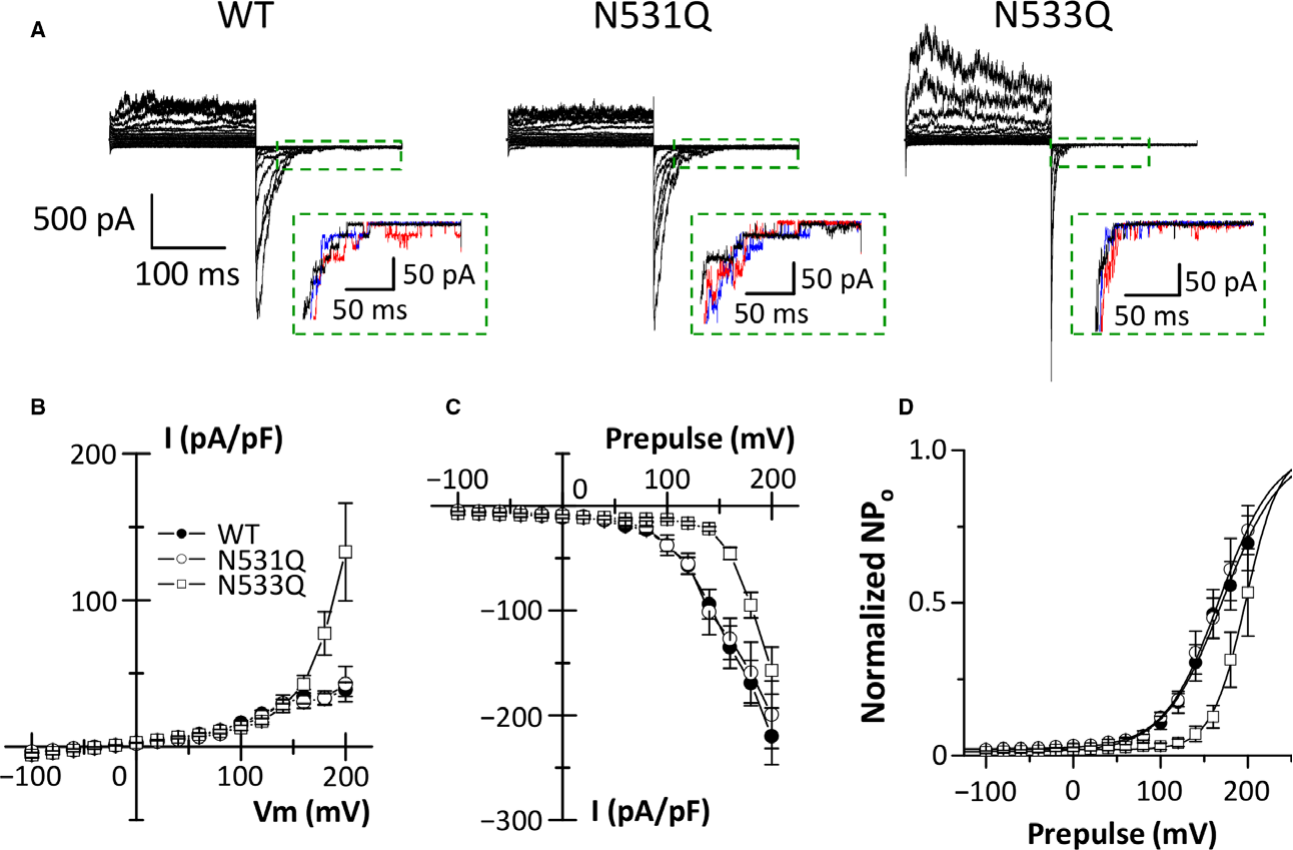
A single mutation, N533Q, of mouse PKD2L1 affects the voltage‐dependent inactivation of the PKD2L1 channel. (A) Representative whole‐cell currents obtained from HEK293T cells expressing the WT channels and the mutants (N531Q and N533Q). Insets: Tail currents at −100 mV evoked by prepulses of +160 mV (black), +180 mV (blue), and +200 mV (red) enclosed in a dashed box are enlarged to show single‐channel currents of the PKD2L1 proteins. (B and C) Current–voltage relationships of steady‐state currents (B) and instantaneous tail currents (C) of the WT channels (closed circles) and the mutants (N531Q: open circles, N533Q: open squares). (D) Activation curves for the WT channels and the mutants. Solid lines represent fits with the Boltzmann equation. The data are averaged from four to eight experiments.

We further investigated which of the two asparagine residues, N531 or N533, affects the voltage‐dependent inactivation of the PKD2L1 channel. To generate the single PKD2L1 mutants, each asparagine residue was replaced with glutamine instead of an alanine, because the side chain of the glutamine is longer than that of asparagine. Thereby, we tried to examine an appropriate length of the side chain at positions 531 and 533 in the voltage‐dependent inactivation of the PID2L1 channels. The N531Q mutants exhibited PKD2L1 currents similar to the WT channels (Fig. 3A–C). The *V*_half_ and the slope factor in the activation curve of the N531Q mutants were 169.7 ± 10.7 mV (*P *=* *0.62) and 30.3 ± 3.3 mV (*P *=* *0.46), respectively (Fig. 3D). On the other hand, the N533Q mutants surprisingly showed larger outward currents upon depolarization and a faster decay of tail currents compared with the WT channels (Fig. 3A,B). As with the double mutants, voltage dependence of the tail currents of the N533Q mutants was different from that of the WT channels (Fig. 3C). The N533Q mutation shifted the activation curve to more positive potentials and decreased its slope factor (Fig. 3D). The activation curve of the N533Q mutant activity had a *V*_half_ of 204.6 ± 16.5 mV (*P *=* *0.02) and a slope factor of 18.6 ± 2.1 mV (*P *=* *0.04). The single‐channel current amplitudes in the N531Q and N533Q mutants were −19.8 ± 0.4 pA (*P *=* *0.40) and −19.9 ± 0.8 pA (*P *=* *0.50), respectively. These results suggest that the asparagine residue at position 533 of the PKD2L1 channel regulates the channel inactivation upon depolarization.

### The N533 mutant of the mouse PKD2L1 channel lacks an increase in the channel activity after the reversal of alkalization and heating

We have previously demonstrated that heating and a shift to alkaline pH at the extracellular side regulate the voltage‐dependent inactivation of the PKD2L1 channel, thereby causing a marked increase in the channel currents during a subsequent decrease in pH and temperature 5, 6. We therefore investigated whether the mutations of the PKD2L1 channel affect its alkali and temperature sensitivity.

**Figure 4:**
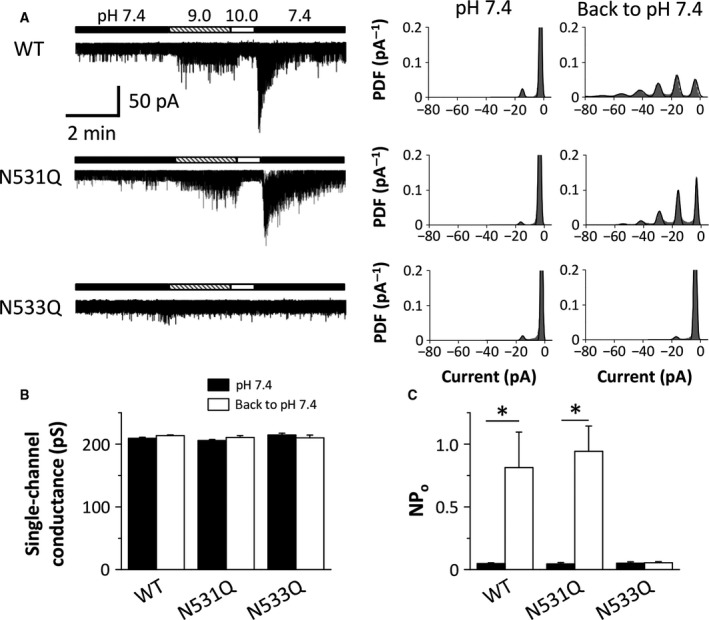
The sensitivity of the mouse PKD2L1 channel to alkalization. (A) Representative whole‐cell PKD2L1 currents recorded at −60 mV in HEK293T cells expressing the WT channels and the mutants (N531Q and N533Q). The pH in the bathing solutions was changed as indicated by the horizontal bars. The corresponding all‐point amplitude histograms at pH 7.4 before and immediately after alkalization are shown on the right. PDF: probability density function. (B and C) The single‐channel conductance (B) and the channel activity (NPo) (C) of the WT channels and the mutants at pH 7.4 before and after alkalization. The data are averaged from three to five experiments. *P <* *0.05.

Single‐channel PKD2L1 currents can be observed at a holding potential of −60 mV in whole‐cell patch‐clamp recordings of PKD2L1‐expressing cells 4, 5, 6. As shown in Fig. 4A, the WT channels exhibited a constitutive activity, which increases and then decreases as extracellular pH is increased. Consistent with our previous findings 5, subsequent neutralization to pH 7.4 caused transient activation of the PKD2L1 channels (Fig. 4A). The current change is dependent on the shift of the voltage‐dependent activation curve. The N531Q and N533Q mutants had a similar basal activity to the WT channels (Fig. 4A). The single‐channel conductance and channel activity (NPo) of these mutants were comparable to those of the WT channels (Fig. 4B,C). On the other hand, the N531Q mutants were activated by a pH drop after alkalization, whereas the N533Q mutants were not (Fig. 4A). Single‐channel analysis demonstrated that the N533Q mutation influenced the channel activity (NPo), but not the single‐channel conductance (Fig. 4B,C).

**Figure 5:**
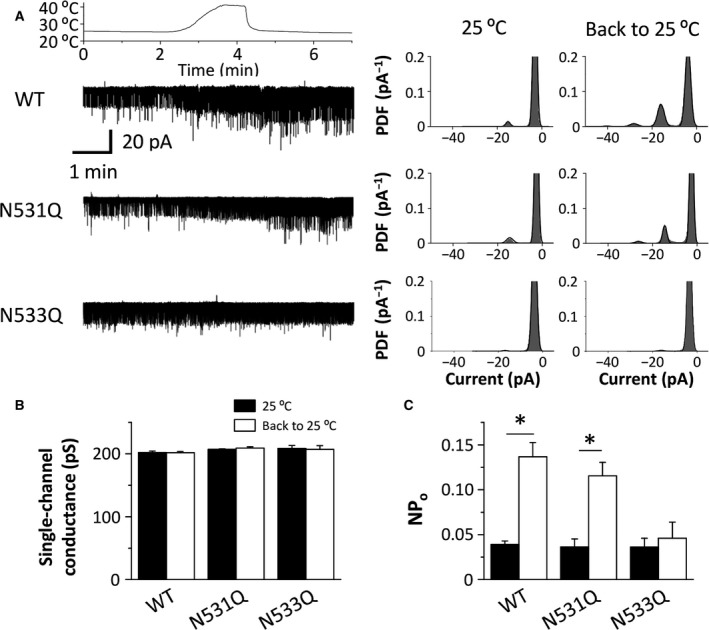
The temperature sensitivity of the mouse PKD2L1 channel. (A) Representative whole‐cell PKD2L1 currents recorded at −60 mV in HEK293T cells expressing the WT channels and the mutants (N531Q and N533Q). The temperature in the bathing solution was changed as shown in the upper panel. The corresponding all‐point amplitude histograms at 25 °C before and after heating are shown on the right. PDF: Probability density function. (B and C) The single‐channel conductance (B) and the channel activity (NPo) (C) of the WT channels and the mutants at 25 °C before and after heating. The data are averaged from four experiments. *P <* *0.05.

We next examined the temperature sensitivity of the PKD2L1 channel. As previously reported 6, although heating to 40 °C increased and decreased a single‐channel conductance and channel activity (NPo) of the WT channels, respectively (data not shown), rapid decrease in the temperature induced an obvious activation of the WT channels (Fig. 5A). The N531Q mutants showed activation by a temperature drop similar to that of the WT channels (Fig. 5A). On the other hand, the N533Q mutants did not induce such a current activation (Fig. 5A). In contrast to the WT channels and N531Q mutants, the N533Q mutants lacked the increase in the channel activity (NPo) during cooling (Fig. 5C), although the single‐channel conductance was not altered by the mutations (Fig. 5B). These results suggest that the N533 residue of the PKD2L1 channel also regulates its sensitivity to alkalization and heating.

## Discussion

Voltage‐dependent inactivation of ion channels is crucial in controlling the membrane excitability of excitable cells. In the present study, we focused on the outer pore loop region of the PKD2L1 channel to investigate its inactivation mechanism, because it is well known that the region modulates C‐type inactivation of voltage‐dependent K^+^ channels such as the HERG K^+^ channel 8, 9, 10, 12, 21, 22. We here identified a key amino acid residue for voltage‐dependent inactivation of the mouse PKD2L1 channel. While the WT PKD2L1 channels exhibited small outward currents upon depolarization, the outward currents of the N533Q mutants were much larger (Fig. 3A,B). The increase in outward currents in the N533Q mutants is due to lack of the transition from open to inactivated states during depolarization, because we could not observe similar tail currents in the N533Q mutants to that in the WT channels. Decay of the tail currents in the N533Q mutants was much faster than that in the WT channels (Fig. 3A). In addition, the voltage dependence of the tail currents in the N533Q mutants was clearly different from that in the WT channels (Fig. 3C). The N533Q mutants had a rightward shift and a steeper slope of the activation curve compared with the WT channels (Fig. 3D). Importantly, the tail currents of N533Q mutants were observed only upon repolarization from depolarized potentials that showed the large outward currents (Fig. 3B,C). These results suggest that the N533Q mutants activate, but do not subsequently inactivate, upon depolarization and that the following repolarization causes its deactivation (the transition process from open to closed states). On the other hand, although the single‐channel conductance and the basal channel activity (NPo) of the N533Q mutants were similar to those of the WT channels (Figs 4 and 5), the gating property of the N533Q mutants seems to be different from that of the WT channels. The WT channels showed long opening events in the decaying process of the tail currents, whereas the N533Q mutants displayed flickering events (Fig. 3A inset). Further studies are awaited to investigate the gating difference between the WT PKD2L1 channel and the N533Q mutant.

C‐type inactivation in voltage‐dependent K^+^ channels is considered to involve structural changes around the selectivity filter 9, 10, 22. Actually, the ‘open‐inactivated’ structure of the *Streptomyces lividans* K^+^ channel, KcsA, has been crystallized and proposed as a model of C‐type inactivation 11. The structure revealed that conformation changes in the selectivity filter mainly restrict ion occupancy in the filter, although the backbone of the outer pore loop region shows only a modest conformational rearrangement. In addition, it has been recently reported that the transition between the inactivated and conductive states of the KcsA channel is accompanied by changes in local outer vestibule dynamics without great conformational changes 23. These results suggest that the local structural dynamics of the outer pore loop region are involved in C‐type inactivation of the KcsA channel. In the present study, we demonstrated that the asparagine residue at position 533 (N533) modulates the voltage‐dependent inactivation of the PKD2L1 channel. The outer pore loop region of the mouse PKD2L1 channel may contribute to the voltage‐dependent inactivation, in consistent with C‐type inactivation of these voltage‐dependent K^+^ channels.

Although the outer pore loop region is considered to be involved in C‐type inactivation of voltage‐dependent K^+^ channels, the mechanism of inactivation remains obscure. A model in which the selectivity filter is dilated on the extracellular side during C‐type inactivation, however, has been recently proposed 22. The dilation changes K^+^ occupancy at the outer side of the selectivity filter, and results in C‐type inactivation. At present, the detailed gating mechanism of the PKD2L1 channel is unknown. Quite recently, the three‐dimensional structure of human PKD2, which is an isoform of PKD2L1, has been revealed 24, 25, 26. In the structural analysis, the PKD2 channel has a tetrameric architecture similar to many voltage‐gated K^+^ channels 27, 28 and TRP channels 29, 30, 31. However, the large extracellular domain between transmembrane segments 1 and 2 (termed polycystin domain 24 or TOP domain 25, 26) is unique to the PKD2 channel. The extracellular domain covers the pore without blocking the ion‐conducting pathway. An aspartate residue (D523) within the selectivity filter in the human PKD2L1 channel has been previously demonstrated to be responsible for Ca^2+^‐dependent inactivation of the channel 32. Consistently, the PKD2 structure in the multi‐ion mode revealed that Ca^2+^ ion is bound to a high‐affinity site formed by the conserved aspartate residue (N643) in the selectivity filter of PKD2, suggesting that the binding of Ca^2+^ ion into the entrance of the selectivity filter might contribute to Ca^2+^‐dependent inactivation 26. On the other hand, we have here demonstrated that mutation of an asparagine residue (N533Q) in the outer pore loop region of the PKD2L1 channel modified its voltage‐dependent inactivation. The extracellular domain (between transmembrane segments 1 and 2) of PKD2 directly interacts with its outer pore loop region and the interaction is considered to regulate the channel gating. Interestingly, the asparagine residue (N653) in the outer pore loop region of PKD2 corresponding to the N533 residue of PKD2L1 [see Fig. 2 in 24, Fig. S1 in 25] is demonstrated to be important for the formation of a tight tripartite complex with the extracellular domain (between transmembrane segments 1 and 2) and membrane lipids in single‐ion mode of the PKD2 structure 26. Therefore, this interaction through the N533 residue of PKD2L1 might regulate the voltage‐dependent inactivation. In the present study, replacement of the asparagine residue (N533) in PKD2L1 with alanine or glutamine lacked the voltage‐dependent inactivation (Figs 2 and 3). Judging from properties of the side chains, proper length rather than functional similarity of the side chains is suggested to be essential for the voltage‐dependent inactivation. The length difference in the side chains might trigger deformation of the tripartite complex in the PKD2L1 channel. It is intriguing that the width of the selectivity filter of PKD2 is changed in the state‐dependent manner 26. Similar to C‐type inactivation of the voltage‐dependent K^+^ channels, it seems possible that a small dilation of the selectivity filter by changing the interaction of the extracellular domain (between transmembrane segments 1 and 2) with the outer pore loop region might cause the voltage‐dependent inactivation of the PKD2L1 channel.

We also demonstrated that the N533Q mutants, in contrast to the N531Q mutants, showed no pH drop‐ or temperature drop‐induced increase in channel activity (Figs 4 and 5). We consider that no changes in the channel activity in the N533Q mutants are due to lack of the voltage‐dependent inactivation. It is currently unknown how extracellular alkalization and heating affect the voltage‐dependent inactivation of the PKD2L1 channels. However, it could be possible that structural changes in the large extracellular domain (between transmembrane segments 1 and 2) triggered by extracellular alkalization and heating allosterically regulate the gating of the PKD2L1 channels through the interaction between the extracellular domain and the outer pore loop region.

The present study suggests that local structural changes in the outer pore loop region of the PKD2L1 channel regulate its voltage‐dependent inactivation. This is the first report showing that the N533 residue in the outer pore loop region contributes to the voltage‐dependent inactivation of PKD2L1, a member of the TRP channel family. Most recently, an important role for the PKD2L1 channel in spinal cerebrospinal fluid contacting (CSF‐c) neurons of the mouse brainstem has been reported. The activity of PKD2L1 channels expressed in CSF‐c neurons regulates their excitability, suggesting that the PKD2L1 channel acts as a spike generator 33. In addition, the CSF‐c neurons have a sensory function being capable of sensing the movement and composition of CSF. Intriguingly, extracellular alkalization has been demonstrated to increase the firing frequency of the CSF‐c neurons, in which firing is mediated by the activity of the PKD2L1 channel 33, 34. Therefore, it would be interesting to investigate how the voltage‐dependent inactivation of the PKD2L1 channels contributes to the sensory functions of the CSF‐c neurons.

## Author contributions

TS, TH, and HS designed experiments. TS, TH, TT, CO, and TF performed experiments. TS, TH, and TF analyzed data. TS, TH, BN, and HS wrote the manuscript.
